# PHB2 Alleviates Neurotoxicity of Prion Peptide PrP^106–126^ via PINK1/Parkin-Dependent Mitophagy

**DOI:** 10.3390/ijms242115919

**Published:** 2023-11-02

**Authors:** Xiaohui Zheng, Kun Liu, Qingqing Xie, Hangkuo Xin, Wei Chen, Shengyu Lin, Danqi Feng, Ting Zhu

**Affiliations:** 1 Key Laboratory of Fujian-Taiwan Animal Pathogen Biology, College of Animal Sciences, Fujian Agriculture and Forestry University, Fuzhou 350002, Chinaliukun5555@yeah.net (K.L.); x1184304548@163.com (Q.X.);; 2 Key Laboratory of Animal Pathogen Infection and Immunology of Fujian Province, College of Animal Sciences, Fujian Agriculture and Forestry University, Fuzhou 350002, China

**Keywords:** mitophagy, prion peptide, PrP^106–126^, PHB2, PINK1/Parkin, neuronal death, prion disease

## Abstract

Prion diseases are a group of neurodegenerative diseases characterized by mitochondrial dysfunction and neuronal death. Mitophagy is a selective form of macroautophagy that clears injured mitochondria. Prohibitin 2 (PHB2) has been identified as a novel inner membrane mitophagy receptor that mediates mitophagy. However, the role of PHB2 in prion diseases remains unclear. In this study, we isolated primary cortical neurons from rats and used the neurotoxic prion peptide PrP^106–126^ as a cell model for prion diseases. We examined the role of PHB2 in PrP^106–126^-induced mitophagy using Western blotting and immunofluorescence microscopy and assessed the function of PHB2 in PrP^106–126^-induced neuronal death using the cell viability assay and the TUNEL assay. The results showed that PrP^106–126^ induced mitochondrial morphological abnormalities and mitophagy in primary cortical neurons. PHB2 was found to be indispensable for PrP^106–126^-induced mitophagy and was involved in the accumulation of PINK1 and recruitment of Parkin to mitochondria in primary neurons. Additionally, PHB2 depletion exacerbated neuronal cell death induced by PrP^106–126^, whereas the overexpression of PHB2 alleviated PrP^106–126^ neuronal toxicity. Taken together, this study demonstrated that PHB2 is indispensable for PINK1/Parkin-mediated mitophagy in PrP^106–126^-treated neurons and protects neurons against the neurotoxicity of the prion peptide.

## 1. Introduction

Prion diseases are infectious neurodegenerative disorders characterized by vacuolar degeneration of the central nervous system (CNS). They affect various animal species and have limited zoonotic potential [1,2,3]. The transmissible nature of prion diseases is attributed to the template-directed misfolding of the normal cellular prion protein (PrP^C^) by the disease-associated conformation (PrP^Sc^) [4,5,6]. This misfolding event leads to increased protease resistance and β-sheet content in the protein, resulting in the deposition of PrP^Sc^ in the CNS [7,8]. The neurotoxic PrP fragment 106–126 (PrP^106–126^) shares many physiological properties and pathogenic characteristics with PrP^Sc^. Consequently, PrP^106–126^ is commonly used to investigate the structural and physicochemical features underlying PrP neurotoxicity [9,10,11].

The mitochondrion plays a critical role in the life of eukaryotic cells and is involved in regulating cell death, innate immune responses, and cell differentiation [12,13]. In prion diseases, mitochondrial dysfunction is frequently observed in brain tissues [9,14,15]. Damaged mitochondria are impaired in ATP production and release higher levels of reactive oxygen species (ROS) [16,17], which are detrimental to cells. Consequently, the autophagic system targets impaired mitochondria for degradation through a catabolic process known as mitophagy, which contributes to maintaining mitochondrial quality control in various cell types [18,19,20].

Two key mitochondrial proteins, PINK1 (PTEN-induced kinase 1) and PRKN/Parkin (parkin RBR E3 ubiquitin protein ligase), regulate the activity of mitophagy [21,22,23]. PINK1, a serine/threonine kinase located on mitochondria, serves as a molecular sensor of mitochondrial health [24,25]. It continuously monitors mitochondrial status and detects damage, signaling for the recruitment of Parkin into depolarized mitochondria [26,27]. Once recruited, Parkin conjugates ubiquitin onto proteins of the outer mitochondrial membrane (OMM), thereby facilitating the elimination of damaged mitochondria through mitophagy [28]. The PINK1/Parkin pathway acts as a crucial amplifying mechanism, enhancing the efficiency of mitophagy. Mutations in this pathway contribute to the pathogenesis of neurodegenerative diseases [29].

Prohibitin 2 (PHB2) is a highly conserved inner mitochondrial membrane (IMM) protein that is critical in regulating mitochondrial assembly and function [30]. Previous studies have shown that PHB2 acts as a key mitophagy receptor in mammalian cells, facilitating Parkin-mediated mitophagy by stabilizing PINK1 and enhancing the recruitment of Parkin to mitochondria [31,32]. Furthermore, PHB2 mediates mitophagy by interacting with LC3, a protein associated with the autophagosomal membrane, and binding to damaged mitochondria through its LC3-interaction region domain [30]. However, the specific impact of PHB2 on mitophagy in the context of prion diseases remains unclear.

In this study, we aimed to examine the status of mitophagy and the involvement of PHB2 in mitophagy and neuronal death in primary cortical neurons treated with PrP^106–126^. Our findings demonstrated that PrP^106–126^ induced PINK1/Parkin-dependent mitophagy in primary neurons. We also determined that PHB2 is essential for PINK1/Parkin-mediated mitophagy and plays a protective role against the neurotoxic effects of the prion peptide on neurons.

## 2. Results

### 2.1. PrP^106–126^ Caused Morphological Abnormality of Mitochondria in the Primary Neurons

**Figure 1:**
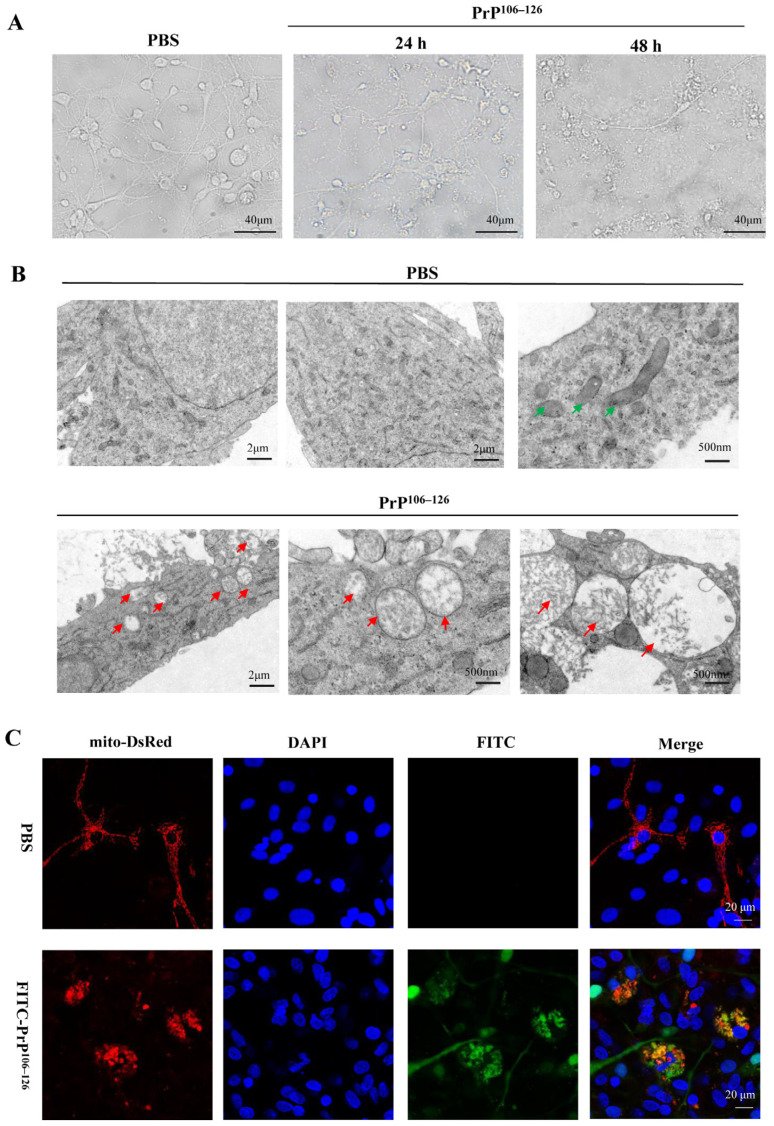
PrP^106–126^ caused morphological abnormality of mitochondria in primary neurons. (**A**) Cortical neurons were treated with PrP^106–126^ for 24 h and 48 h, respectively, and then the morphologic changes of neurons were observed using light microscope. (**B**) Mitochondrial ultrastructure observation of cortical neurons treated with PrP^106–126^ for 24 h using TEM. Green arrows indicate healthy mitochondria; red arrows indicate swollen mitochondria. (**C**) Primary neurons were incubated with 25 μM FITC-labeled PrP^106–126^ (FITC-PrP^106–126^) for 24 h; the colocalization between FITC-PrP^106–126^ (green) and mitochondria (labeled with mito-DesRed, red) in cortical neurons were observed by immunofluorescence imaging (nuclei, blue).

Neuronal mitochondria play a critical role in maintaining cellular homeostasis, modulating reactive species, providing energy in the form of ATP through oxidative phosphorylation, and regulating various forms of programmed cell death, among numerous other functions [33,34,35]. Previous studies have described the dysfunction and morphological changes of mitochondria in the brains of prion diseases [9,14]. In our study, primary neurons were treated with 100 μM PrP^106–126^ for 24 h and 48 h, respectively, and then the morphology changes of the neurons were observed. As shown in Figure 1A, PrP^106–126^-treated neurons exhibited neuron shrink in size, and some of them were lost. The axons were broken, fragmented, or even disappeared. Subsequently, we investigated the ultrastructural changes of mitochondria; primary cortical neurons were treated with 100 μM PrP^106–126^ for 24 h and then fixed and subjected to TEM observations. In the TEM images of the control group (Figure 1B, top panel), mitochondria with clear cristae and matrix were evident. In contrast, in neurons exposed to PrP^106–126^ stimulation (Figure 1B, bottom panel), many mitochondria became swollen and rounder, the matrix became shallower, and the cristae fractured or even disappeared. These findings suggest that PrP^106–126^ induces severe morphological abnormalities in mitochondria.

Aiken and colleagues suggested that the scrapie agent, or prion, was present in brain mitochondria from prion-infected hamsters [36]. To investigate whether PrP^106–126^ was also present in the mitochondria of primary neurons, we transfected mito-DesRed into primary neurons for 24 h, followed by the treatment with FITC-PrP^106–126^ for an additional 24 h. The colocalization between mitochondria and FITC-PrP^106–126^ was examined. The results demonstrated that mitochondria predominantly colocalized with FITC-PrP^106–126^ in the neuronal cell body (Figure 1C). These findings indicate that PrP^106–126^ is present in mitochondria and may induce mitochondrial morphological abnormalities in primary neurons.

### 2.2. PrP^106–126^ Induced Mitophagy in Primary Neurons

**Figure 2:**
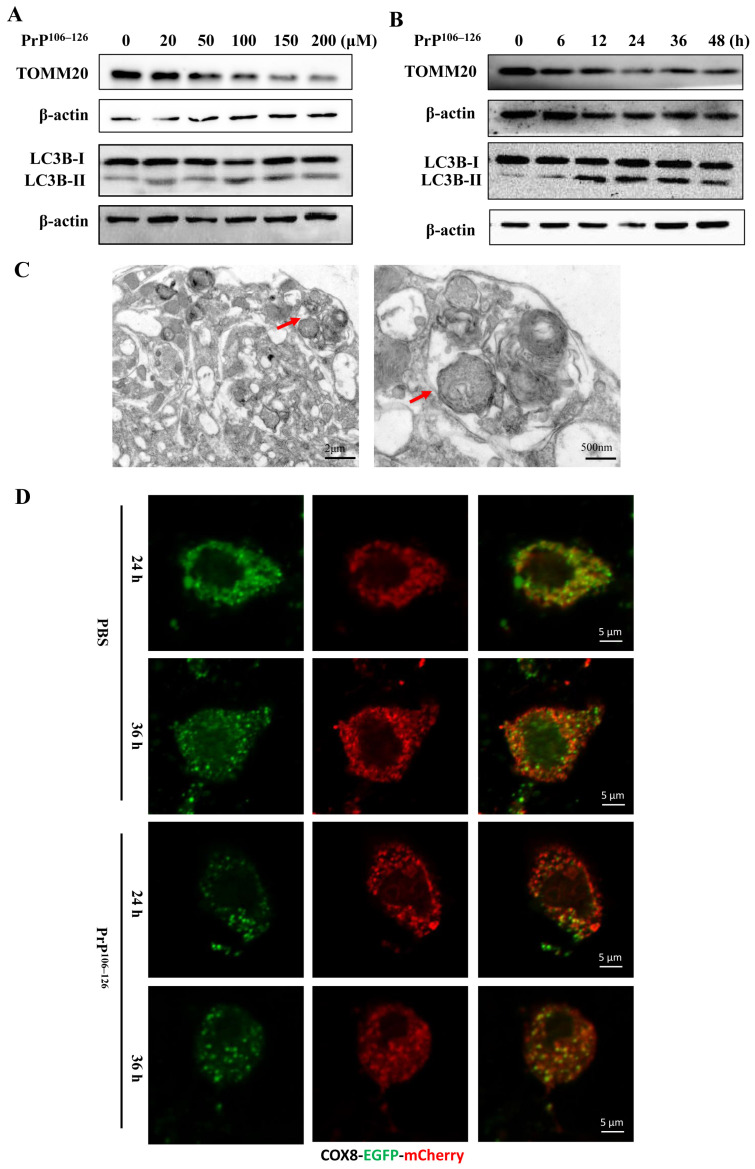
PrP^106–126^ induced mitophagy in primary neurons. (**A**) Cortical neurons were treated with different concentrations of PrP^106–126^ for 24 h, and then, the protein levels of TOMM20 and LC3B-II were analyzed using Western blotting. (**B**) Cortical neurons were treated with PrP^106–126^ for the indicated periods, and the protein levels of TOMM20 and LC3B-II were analyzed by Western blotting. (**C**) Representative TEM images of autophagosome containing mitochondria (red arrows) in PrP^106–126^-treated cortical neurons. (**D**) Mitophagy in cortical neurons transfected with COX8-EGFP-mCherry. After transfection, the cells were subjected to the PrP^106–126^ treatment for 24 h. The red puncta represent mitochondria in lysosomes with acidic pH.

Mitophagy is an evolutionarily conserved process that involves the clearance of damaged mitochondria through the autophagy–lysosome pathway, playing an essential role in maintaining the health of the mitochondrial network [37]. In this study, we examined the occurrence of mitophagy in primary neurons exposed to PrP^106–126^. Primary cortical neurons were treated with different concentrations of PrP^106–126^ for 24 h, and the protein levels of TOMM20 (translocase of the outer mitochondrial membrane 20), a marker for the mitochondrial outer membrane, and microtubule-associated protein 1 light chain 3B-II (LC3B-II), an autophagosomal marker, were examined via Western blotting. As shown in Figure 2A, the protein levels of TOMM20 reduced, while LC3B-II levels increased after 50 μM PrP^106–126^ treatment. We further treated primary neurons with 100 μM PrP^106–126^ at different times, as shown in Figure 2B. The reduced protein levels of TOMM20 and the increased levels of LC3B- II were detected after 12 h treatment (Figure 2B). Additionally, using TEM, we observed larger double-layer membrane structures containing damaged and broken mitochondria in PrP^106–126^-treated neurons (Figure 2C), indicating the induction of mitophagy. To further confirm the induction of mitophagy, we expressed COX8-EGFP-mCherry, a tandem fluorescent-tagged mitochondrial targeting sequence of the inner membrane protein COX8, in primary neurons. This allowed us to monitor the delivery of mitochondria to lysosomes based on the different pH stability of EGFP and mCherry fluorescent proteins. As depicted in Figure 2D, PBS-treated neurons displayed yellow staining of mitochondria with a merge of green (EGFP) and red (mCherry) signals at 24 and 36 h. In contrast, distinct red puncta were detected in PrP^106–126^-treated neurons at 24 and 36 h, indicating an increased delivery of mitochondria into lysosomes. Collectively, these findings suggest that mitophagy is induced in primary neurons in response to the PrP^106–126^ treatment.

### 2.3. PrP^106–126^ Triggers PINK1/Parkin-Dependent Mitophagy

**Figure 3:**
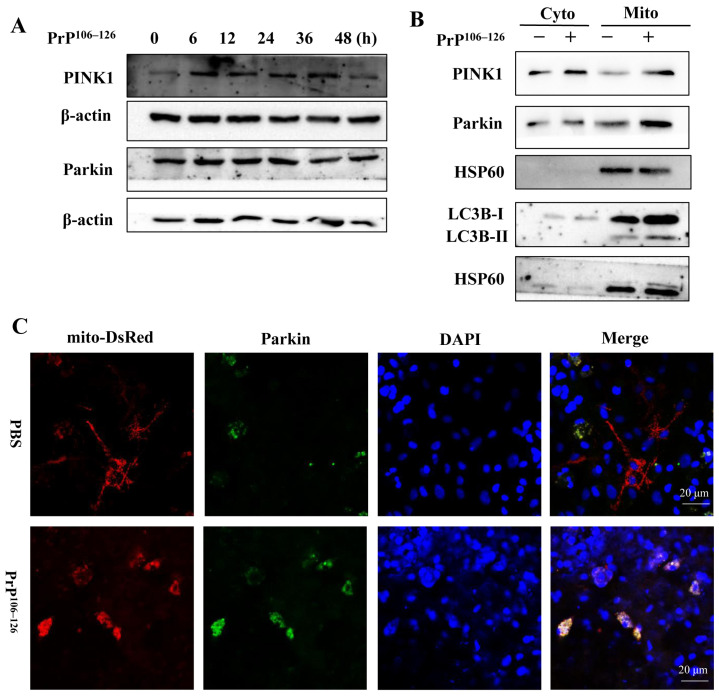
PrP^106–126^ triggers PINK1/Parkin-dependent mitophagy. (**A**) Cortical neurons were treated with PrP^106–126^ for the indicated periods, and then, the protein levels of PINK1 and Parkin were analyzed by Western blotting. (**B**) Proteins of cytoplasm and mitochondria in PrP^106–126^-treated neurons and control neurons were extracted, respectively, and the protein levels of PINK1, Parkin, and LC3B-II were analyzed using Western blotting; HSP60 (the mitochondrial matrix protein) served as loading control. (**C**) The colocalization between mitochondria (labeled with mito-DesRed, red) and Parkin (green) in cortical neurons was observed using immunofluorescence imaging (nuclei, blue).

In healthy cells, PINK1 is continually degraded by mitochondrial proteases. However, upon mitochondrial damage, PINK1 proteolysis is inhibited, leading to its accumulation in the mitochondria. Subsequently, this accumulation promotes the recruitment of cytosolic E3 ubiquitin protein ligase Parkin to the mitochondrial outer membrane, facilitating the process of mitophagy [26,38]. PINK1/Parkin-dependent mitophagy is an extensively studied form of mitophagy and has been implicated in the pathogenesis of neurodegenerative diseases [39]. To investigate the involvement of PINK1 and Parkin in PrP^106–126^-induced mitophagy, primary neurons were exposed to PrP^106–126^ for 6 to 48 h, and the protein levels of PINK1 and Parkin were examined via Western blotting analysis. As depicted in Figure 3A, the PrP^106–126^ treatment increased the protein level of PINK1, followed by a slight increase in the Parkin protein level.

Moreover, cytoplasmic and mitochondrial fractions were extracted from PrP^106–126^-treated neurons and control neurons, respectively, and subjected to Western blotting. The results displayed elevations in the protein levels of PINK1, Parkin, and LC3B-II in the mitochondria of PrP^106–126^-treated neurons compared to the non-treated group (Figure 3B). Additionally, mito-DesRed, a eukaryotic expression plasmid that is used as a mitochondrial marker, was transfected into primary neurons for 24 h, followed by the treatment with PrP^106–126^. Confocal microscopy was employed to examine the colocalization between mitochondria and Parkin. As shown in Figure 3C, the PrP^106–126^ treatment increased the merging of green (Parkin) and red (mitochondria) fluorescence, indicating the recruitment of Parkin to mitochondria. In summary, these findings suggest that PrP^106–126^ treatment increases the protein level of PINK1 and stabilizes PINK1 in the mitochondrial fraction, promoting the recruitment of Parkin to mitochondria and mediating mitophagy in primary neurons.

### 2.4. PHB2 Involves in PrP^106–126^-Induced Mitophagy in Primary Neurons

**Figure 4:**
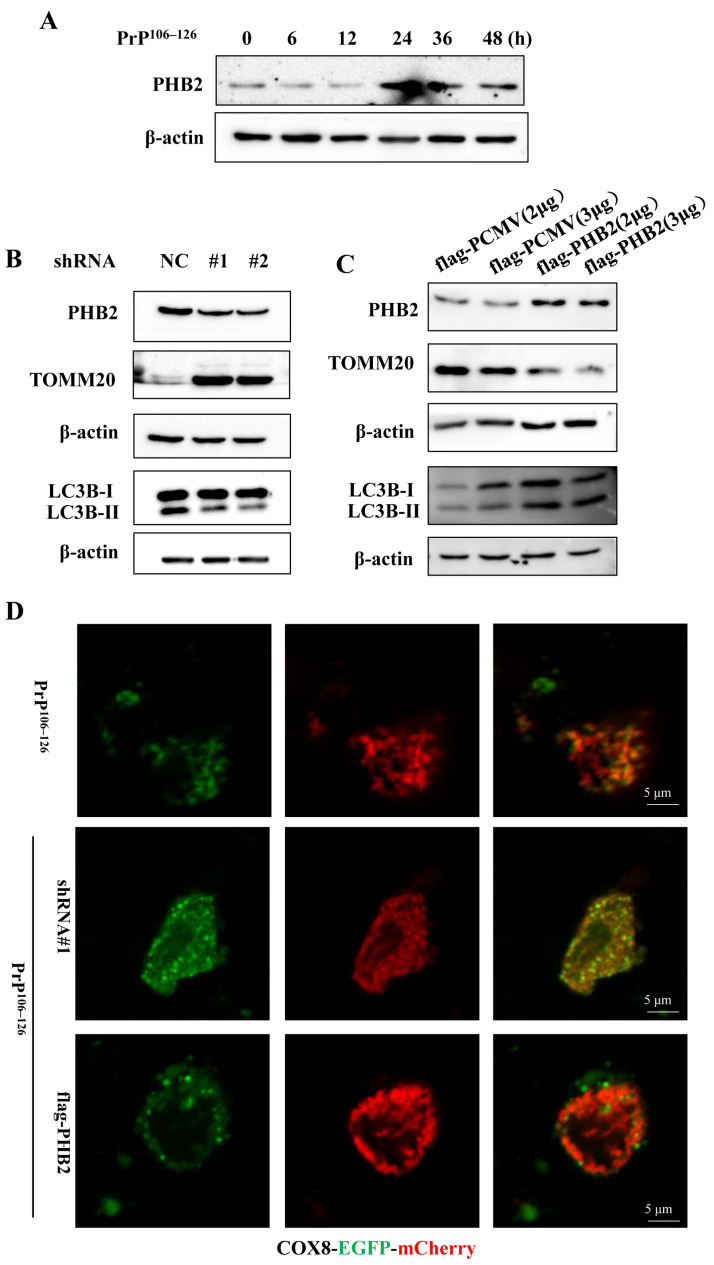
PHB2 involves PrP^106–126^-induced mitophagy in primary neurons. (**A**) Cortical neurons were treated with PrP^106–126^ for the indicated periods, and then, the protein levels of PHB2 were analyzed using Western blotting. (**B**) Cortical neurons were transfected with shRNA-PHB2 (sh#1, sh#2) plasmids or shRNA-NC control plasmid before the incubation with PrP^106–126^ for 24 h. PHB2, TOMM20, and LC3B-II were analyzed using Western blotting. (**C**) Cortical neurons were transfected with different concentrations of flag-PHB2 vector or flag-PCMV control vector for 24 h and then incubated with PrP^106–126^ for another 24 h. PHB2, TOMM20, and LC3B-II proteins were analyzed using Western blotting, respectively. (**D**) Representative images of PrP^106–126^-induced mitophagosome (red) formation in PHB2-knockdown neurons or PHB2-overexpression neurons. Cortical cells were firstly transfected with shRNA-PHB2 (sh#1) and flag-PHB2, respectively, and 24 h later, these neurons were transfected with COX8-EGFP-mCherry. After 24 h, the neurons were subjected to the PrP^106–126^ treatment for another 24 h. Finally, the neurons were fixed for confocal microscopy.

Prohibitin 2 (PHB2), an essential IMM protein, serves as a receptor in mitophagy and is responsible for mitochondrial quality control [31]. Our findings demonstrate that the PrP^106–126^ treatment increases the protein level of PHB2 (Figure 4A). To investigate the involvement of PHB2 in PrP^106–126^-induced mitophagy, different shRNAs were transfected into primary neurons to knock down endogenous PHB2, and the efficiency of knockdown was confirmed by Western blotting (Figure 4B). After treating the neurons with PrP^106–126^ for 24 h, the protein levels of LC3B-II and TOMM20 were examined. As shown in Figure 4B, compared to the control group transfected with shRNA-NC, the PHB2-knockdown groups exhibited a reduction in LC3B-II levels, while the protein level of TOMM20 increased. Additionally, we transfected primary neurons with different concentrations of the recombinant vector flag-PHB2 for 24 h, using the empty vector flag-PCMV as a control. Subsequently, the neurons were treated with PrP^106–126^ for an additional 24 h. As shown in Figure 4C, the PHB2-overexpression group exhibited an increase in LC3B-II and a decrease in TOMM20 protein levels, indicating that PHB2 may participate in PrP^106–126^-induced mitophagy. To further verify the influence of PHB2 on mitophagy, we transfected COX8-EGFP-mCherry into PHB2-knockdown neurons and PHB2-overexpression neurons, respectively. Subsequently, the neurons were exposed to PrP^106–126^ stimulation. The results demonstrated that compared to the peptide-only treatment group, the knockdown of PHB2 increased the yellow staining of mitochondria, indicating the merge of green (EGFP) and red (mCherry) signals. Conversely, the overexpression of PHB2 exhibited more prominent red puncta (Figure 4D), suggesting that PHB2 influences the delivery of mitochondria into lysosomes. Collectively, these results suggest that PHB2 is indispensable for PrP^106–126^-induced mitophagy in primary neurons.

### 2.5. PHB2 Is Required for PINK1/Parkin-Dependent Mitophagy in PrP^106–126^-Treated Neurons

**Figure 5:**
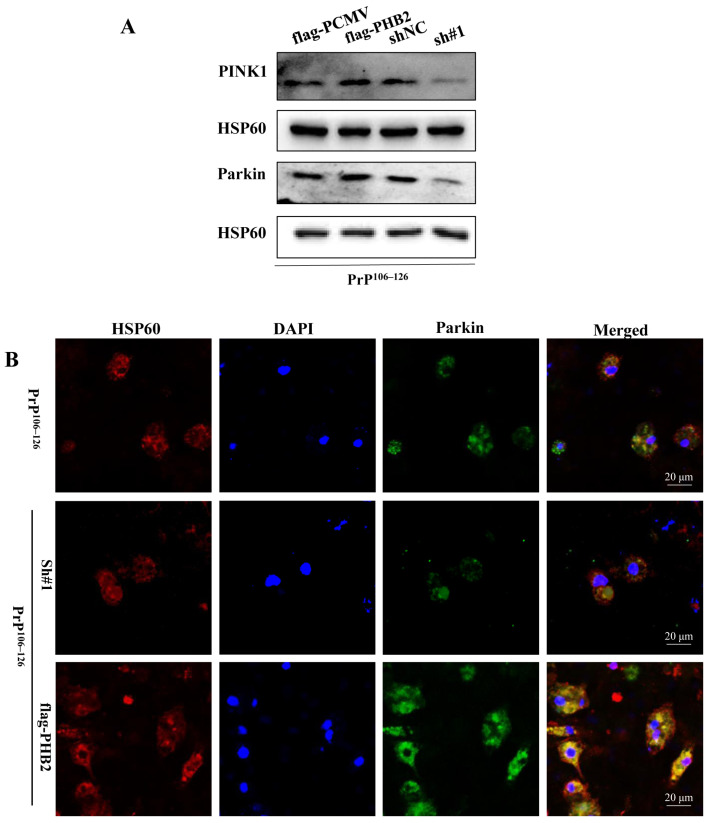
PHB2 is required for PINK1/Parkin-mediated mitophagy in PrP^106–126^-treated neurons. (**A**) Western blotting of PINK1 and Parkin proteins in mitochondria of PHB2-overexpression and PHB2-knockdown neurons with the PrP^106–126^ treatments (HSP60 served as loading control). (**B**) The colocalization between mitochondria (labeled with HSP60, red) and Parkin (green) in PHB2-knockdown and PHB2-overexpression neurons under the PrP^106–126^ treatment was observed using immunofluorescence imaging (nuclei, blue).

Previous studies have demonstrated the crucial role of PHB2 in regulating PINK1/Parkin-mediated mitophagy in mammalian cells [31,39]. Therefore, we extracted mitochondrial proteins from PHB2-knockdown neurons and PHB2-overexpression neurons treated with PrP^106–126^. The Western blotting results revealed that the knockdown of PHB2 reduced the protein levels of PINK1 and Parkin in mitochondria compared to neurons treated with PrP^106–126^ alone. Conversely, the overexpression of PHB2 increased the protein levels of PINK1 and Parkin in mitochondria (Figure 5A).

To assess the colocalization of Parkin with mitochondria, we treated PHB2-knockdown and PHB2-overexpression neurons with PrP^106–126^ for 24 h and performed a confocal microscopy assay. The results showed that PHB2-knockdown neurons displayed fewer yellow stains with the merge of green (Parkin) and red (mitochondria) signals; in contrast, PHB2-overexpression neurons exhibited more yellow fluorescence with the merge of Parkin and mitochondria signals (Figure 5B), which indicate that PHB2 influences the colocalization of Parkin with mitochondria. Therefore, these findings suggest that PHB2 is essential for accumulating PINK1 and subsequent recruitment of Parkin to mitochondria.

### 2.6. PHB2 Modulates PrP^106–126^-Induced Neuronal Death

**Figure 6:**
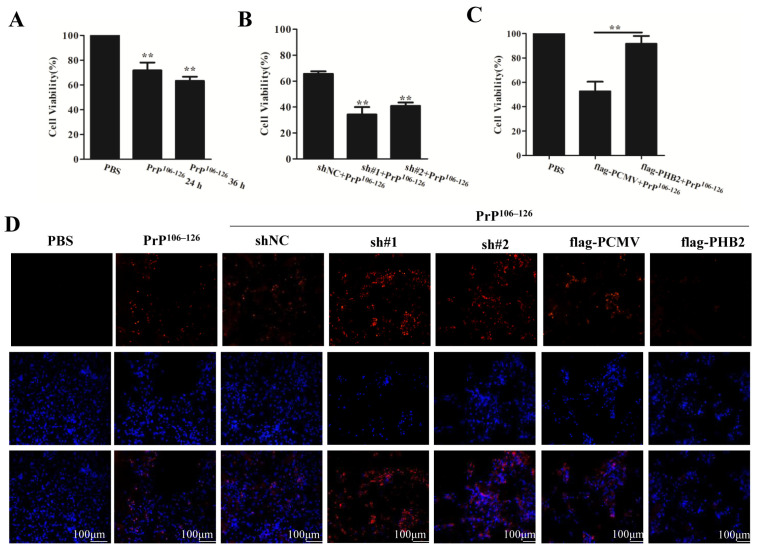
PHB2 attenuates PrP^106–126^-induced neuronal death. Primary neurons (**A**) or PHB2-knockdown neurons (**B**) or PHB2-overexpression neurons (**C**) were treated with PrP^106–126^, the cell viability were measured by using the CCK-8 assay. Bars represent means ± SD (*n* = 3). ** *p* < 0.01 indicate significant differences between the control groups. (**D**) Fluorescence images of cell apoptosis were detected using TUNEL assays (red) in control or treated cortical neurons (nuclei, blue).

Neuronal death is a prominent characteristic of prion diseases, as both PrP^Sc^ and PrP^106–126^ have been shown to induce apoptosis in neuronal cells [40]. Initially, we treated primary neurons with 100 μM PrP^106–126^ for 24 and 36 h, respectively. Cell viability was assessed using the CCK-8 assay, and the results demonstrated that the PrP^106–126^ treatment reduced cell viability compared to PBS-treated neurons at both time points (Figure 6A).

To investigate the role of PHB2 in neuronal survival, we examined the effects of PHB2 knockdown on cell viability in neurons exposed to PrP^106–126^. The results revealed that PHB2 knockdown led to a significant decrease in cell viability compared to the sh-NC control group under PrP^106–126^ stimulation (Figure 6B). Furthermore, we transfected primary neurons with flag-PHB2 and subsequently treated them with PrP^106–126^. As depicted in Figure 6C, the cell viability of flag-PHB2-transfected neurons was markedly higher than that of the flag-PCMV control group, indicating that PHB2 offers some level of protection to neurons against PrP^106–126^ toxicity.

To validate the impact of PHB2 on PrP^106–126^-induced neuronal damage, we conducted a TUNEL assay to measure cell apoptosis. The results demonstrated that the treatment with PrP^106–126^ increased the fluorescence intensity (red) in the TUNEL staining at 24 and 36 h. Moreover, the knockdown of PHB2 resulted in a further increase in the number of TUNEL-positive neurons. Conversely, the overexpression of PHB2 reduced the number of TUNEL-positive neurons (Figure 6D). These findings collectively indicate that PHB2 modulates PrP^106–126^-induced neuronal death. The depletion of PHB2 exacerbates the neuronal toxicity of PrP^106–126^, while overexpression of PHB2 alleviates the neuronal toxicity of PrP^106–126^.

## 3. Discussion

Mitochondria play a crucial role in regulating cell death, a significant characteristic of neurodegeneration [12,41,42]. In our study, we observed that PrP^106–126^ induced morphological abnormalities in mitochondria, such as mitochondrial swelling and vacuolation, in primary neurons. Similar mitochondrial abnormalities have been reported in the brains of various neurodegenerative diseases, including prion diseases [14,32,43]. The impaired mitochondrial activity is typically observed at advanced disease stages in both human patients and animal models of neurodegenerative diseases [44]. It has been documented that mitochondrial dysfunction contributes to the formation of Aβ plaques and neurofibrillary tangles, which are defining features of Alzheimer’s disease, and this, in turn, exacerbates mitochondrial defects [45,46]. Li and colleagues reported extensive mitochondrial fragmentation, the collapse of mitochondrial membrane potential (MMP), ATP loss, and cell death in PrP^106–126^-treated N2a cells in vitro and the hamster prion model in vivo [9].

Additionally, we discovered the presence of PrP^106–126^ in mitochondria. Manczak et al., reported that mitochondria serve as direct sites for Aβ accumulation in neurons affected by Alzheimer’s disease, leading to the generation of free radicals and impairment of mitochondrial metabolism during disease development and progression [47]. In a mouse model of Parkinson’s disease, mutant α-synuclein has been detected within mitochondria in specific brain regions, suggesting that it may directly damage mitochondria [48]. Therefore, we hypothesize that the presence of prion peptides in mitochondria could be one of the reasons leading to mitochondrial damage.

Autophagy is a highly conserved process essential for cellular homeostasis and survival [49]. Moreover, selective autophagy forms specifically target damaged organelles, such as mitophagy, which clears dysfunctional mitochondria [50]. Proficient mitophagy responses are crucial for maintaining optimal mitochondrial numbers, preserving energy metabolism, and protecting cells, including neurons, from the harmful effects of damaged mitochondria [19,37,51]. In this study, we found that the PrP^106–126^ treatment induced mitophagy in primary neurons, which aligns with previous studies indicating enhanced mitophagy in prion-infected cultured cells and prion-infected experimental mice [14]. Mitochondrial dysfunction plays a critical role in developing numerous neurodegenerative diseases, and cells have evolved the capacity to limit impairment by activating mitophagy [52]. Multiple lines of evidence suggest that mitophagy mediates neuroprotective effects in certain forms of neurodegenerative diseases and acute brain damage [46,51,53]. Therefore, the activation of mitophagy in PrP^106–126^-treated neurons may contribute to eliminating damaged mitochondria caused by the prion peptide, to some extent, preserving mitochondrial homeostasis and promoting neuronal survival. However, whether the accumulation of PrP^106–126^ in mitochondria directly activates mitophagy or PrP^106–126^ exerts its toxic effects via other pathways leading to mitophagy remains to be further studied.

Furthermore, we observed an increase in the protein levels of PINK1 and Parkin in PrP^106–126^-treated neurons. PINK1 plays a role in mitochondrial maintenance and functions upstream of Parkin [27,54]. It is selectively stabilized by mitochondrial dysfunction, leading to the recruitment of Parkin to the mitochondrial outer membrane [24,55]. This recruitment triggers the ubiquitination of several mitochondrial outer membrane proteins, such as TOMM20, which, in turn, bind specific autophagy receptors such as SQSTM1/p62 [24,56]. Subsequently, LC3B-coated phagophores encapsulate the damaged mitochondria and facilitate their delivery to the lysosome for degradation [22,26,57]. In our study, we observed higher protein levels of PINK1, Parkin, and LC3B-II in the mitochondria of PrP^106–126^-treated neurons compared to untreated neurons. Additionally, the PrP^106–126^ treatment increased the colocalization between Parkin and mitochondria. These findings suggest that the PrP^106–126^ treatment stabilized PINK1 on mitochondria, leading to the recruitment of Parkin to impaired mitochondria. This recruitment process likely involves the involvement of LC3B-II in mediating mitophagy. However, the specific mechanism needs more research.

PHB2 is a highly conserved mitochondrial inner membrane protein that forms the mitochondrial prohibitin complex along with PHB/PHB1 [58,59,60]. It has been reported that PHB2 binds to the LC3 through an LC3-interaction region (LIR) domain upon mitochondrial depolarization and proteasome-dependent outer membrane rupture. This binding is necessary for the clearance of paternal mitochondria [30,31,38]. Therefore, we explored the role of PHB2 in PrP^106–126^-induced mitophagy. We observed an increase in the expression of PHB2, and PHB2 knockdown inhibited mitophagy; conversely, the overexpression of PHB2 increased mitophagy. These findings suggest that PHB2 is essential for PrP^106–126^-induced mitophagy.

It has been reported that PHB2 is required for classic Parkin-induced mitophagy in mammalian cells [38,61,62]. In our study, we found that PHB2 depletion blocked the mitochondrial accumulation of PINK1 and inhibited the recruitment of Parkin to mitochondria. On the other hand, PHB2 overexpression directly increased PINK1 accumulation and subsequent recruitment of Parkin to mitochondria in primary neurons exposed to PrP^106–126^ stimulation. These data demonstrate that PHB2 is essential for PINK1/Parkin-dependent mitophagy in PrP^106–126^-treated neurons. Several studies have reported that PHB2 may be a novel target for diseases associated with mitophagy. Yan and colleagues found that the small molecule compound FL3 could inhibit the function of PHB2, thereby significantly blocking mitophagy and exerting antitumor effects [38]. Furthermore, PHB2 is also required for cholestasis-induced mitophagy via LC3 into the injured mitochondria [30].

Studies have reported that mitochondrial dysfunction and bioenergy deficiency in many neurodegenerative diseases can be alleviated by stimulating PINK1/Parkin-mediated mitophagy [14,27,63]. Considering our observation of PHB2’s involvement in PINK1/Parkin-dependent mitophagy in PrP^106–126^-treated neurons, we investigated whether PHB2 influences the neurotoxicity of PrP^106–126^. We analyzed the effects of PHB2 on neuronal death and apoptosis induced by PrP^106–126^ stimulation. The results showed that PHB2 knockdown exacerbated PrP^106–126^-induced neuronal death and apoptosis. Conversely, PHB2 overexpression inhibited PrP^106–126^-induced neuronal damage. These findings indicate that PHB2 provides protection for neurons against PrP^106–126^ toxicity. Furthermore, considering the previous finding that PHB2 is required for PINK1/Parkin-mediated mitophagy, it is plausible that the neuroprotection provided by PHB2 against PrP^106–126^ toxicity involves, to some extent, PINK1/Parkin-mediated mitophagy. Several studies have demonstrated the protective role of PHB2-mediated mitophagy in various diseases. One study reported that the depletion of PHB2 decreased the interaction between PHB2 and LC3, resulting in reduced mitophagy and exacerbated loss of dopaminergic neurons in a Parkinson’s disease mouse model [64]. Lai and co-workers reported that Rutin, a natural botanical ingredient, attenuated oxidative damage, through PHB2-mediated mitophagy, in MPP^+^-treated SH-SY5Y cells [65]. Artemisinin, known for its powerful antioxidative stress effect, alleviated oxidative damage caused by cerebral ischemia/reperfusion by regulating PHB2-mediated autophagy in the human neuroblastoma SH-SY5Y cell line [66].

**Figure 7:**
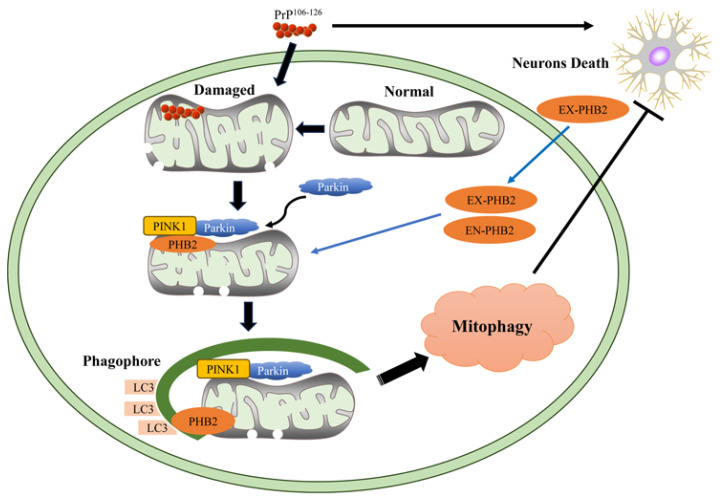
Schematic representation of PHB2-mediated mitophagy and neuronal death inhibition under PrP^106–126^ stimulation. PrP^106–126^ accumulates in mitochondria and leads to mitochondrial damage, which stabilizes PINK1 and recruits Parkin to mitochondria to mediate mitophagy. IMM component PHB2 acts as a mitophagy receptor and plays a vital role in PINK1/Parkin-dependent mitophagy. Endogenous and exogenous PHB2 functions as prosurvival proteins by mediating mitophagy and suppressing neuronal death. EX-PHB2, exogenous PHB2; EN-PHB2, endogenous PHB2.

In conclusion, our findings demonstrate that PrP^106–126^ induces PINK1/Parkin-mediated mitophagy. PHB2, a mitophagy receptor, protects neuronal cells against the toxicity of the prion fragment, and this effect is likely mediated through PINK1/Parkin-dependent mitophagy (Figure 7). While further research is needed to confirm and explore the full role of PHB2 in prion diseases, our studies provide hope for targeting PHB2 as a therapeutic approach for neurodegenerative diseases associated with prions.

## 4. Materials and Methods

### 4.1. Antibodies and Reagents

Rabbit polyclonal anti-HSP60 antibody (A0969), rabbit polyclonal anti-PINK1 antibody (A7131), and mouse monoclonal anti-TOMM20 antibody (A19403) were obtained from ABclonal Technology (Wuhan, China). Mouse monoclonal anti-Parkin antibody (sc-32282) was purchased from Santa Cruz Biotechnology (Santa Cruz, CA, USA). Rabbit polyclonal anti-LC3B antibody (L7543) was obtained from Sigma–Aldrich (St. Louis, MO, USA). Rabbit polyclonal anti-prohibitin 2 (PHB2) (12295-1-AP) antibody, rabbit polyclonal anti-beta actin (β-actin) antibody (20536-1-AP), and mouse monoclonal anti-GAPDH antibody (60004-1-Ig) were obtained from Proteintech Group (Chicago, IL, USA). The goat anti-rabbit HRP-conjugated IgG secondary antibody (ZB-5301) and goat anti-mouse HRP-conjugated IgG secondary antibody (ZB-2305) were purchased from Beijing ZSGB Biotechnology (Beijing, China). Goat anti-rabbit FITC-conjugated IgG secondary antibody (HS111-01), goat anti-mouse FITC-conjugated IgG secondary antibody (HS211-01), and goat anti-rabbit PE-conjugated IgG secondary antibody (HS121-01) were obtained from TransGen Biotech (Beijing, China).

### 4.2. Cell Culture

Cerebral cortex neuronal cultures were prepared from postnatal 1-day-old Sprague-Dawley rats, following the previously described procedure [67,68]. Briefly, after sterilization, the brain was dissected, and then the cerebral cortices were collected and digested with ice-cold HBSS containing 2 mg/mL papain (Solarbio Life Sciences, Beijing, China) and 50 μg/mL DNAse (Sigma–Aldrich, St. Louis, MO, USA) for 30 min at 37°C;. The digested tissues were gently triturated into single cells. After repeated sedimentation and washing, the cells were separated by centrifugation at 800 rpm for 5 min. Then the isolated cells were seeded in 50 μg/mL poly-D-lysine (Solarbio)-coated plates (Corning, Corning, NY, USA) at a final density of 7 × 10^5^ cells/well in a 12-well plate or 5 × 10^5^ cells/well in a 24-well plate. The cells were cultured in DMEM/F12 (Gibco, Grand Island, NY, USA), supplemented with 10% fetal bovine serum (Nulen Biotech, Shanghai, China) and 2% B27 (Invitrogen, Carlsbad, CA, USA). After 48 h, 10 μM cytarabine (Sigma–Aldrich) was added to suppress the growth of glial cells. Experimental treatments were initiated after 6 days of culture.

### 4.3. Prion Protein Peptide and Peptide Treatment

The PrP^106–126^ peptide (sequence: KTNMKHMAGAAAAGAVVGGLG) and fluorescein isothiocyanate-labeled PrP^106–126^ (FITC-PrP^106–126^) were synthesized by Sangon BioTech (Shanghai, China). The purity of the prion peptides was >98%, as indicated by data from the synthesizer. The peptides were dissolved in 0.1 M PBS to a concentration of 1 mM and allowed to aggregate at 37 °C for 24 h [68,69,70]. The neurons were washed with 0.1 M PBS, and then the cells were treated with PrP^106–126^ or FITC-PrP^106–126^ in a culture medium for indicated times.

### 4.4. Mitochondrial Isolation

Mitochondria were isolated using the mitochondrial isolation assay (C3601, Beyotime, Shanghai, China). Briefly, 5 × 10^7^ cortical neurons or treated neurons were washed and resuspended in pre-cooled 0.1 M PBS. The cells were then homogenized with a mitochondrial separation reagent supplemented with a protease inhibitor solution (Beyotime) and then centrifuged at 600× *g* for 10 min at 4 °C. The supernatants were transferred to a clean 1.5 mL tube and centrifuged again at 11,000× *g* for 10 min at 4 °C. The supernatant containing cytoplasmic fractions and the precipitation containing mitochondrial fractions were carefully collected, respectively. The collected cytoplasmic fractions were then centrifuged at 12,000× *g* for 10 min at 4 °C, and the supernatant was collected.

### 4.5. Immunofluorescence Microscopy

Primary cortical neurons were transfected with mito-DesRed and treated after 24 h. The fluorescence images of mitochondria were acquired using a confocal microscope (Carl Zeiss, Oberkochen, Germany).

Primary cortical neurons, PHB2-knockdown neurons, or PHB2-overexpression neurons were transfected with the COX8-EGFP-mCherry plasmid for 24 h, respectively. Subsequently, the neurons were exposed to either PBS or PrP^106–126^. Fluorescence images were visualized using a confocal microscope (Carl Zeiss).

Primary cortical neurons, PHB2-knockdown neurons, or PHB2-overexpression neurons were transfected with mito-DsRed, respectively. The neurons were then fixed, permeabilized, and sealed. Following this, the neurons were incubated with a primary antibody overnight at 4 °C, followed by fluorescently labeled secondary antibodies for 1 h in the dark at 37 °C. Finally, DAPI dihydrochloride was used for nucleus staining, and the fluorescence images were visualized using a confocal microscope (Carl Zeiss).

### 4.6. Plasmids and Transfection

The PHB2 short hairpin RNA (shRNA) expression plasmids shPHB2(#1)(5′-TTCTCCGAACGTGTCACGT-3′), shPHB2(#2) (5′-CTGGACGATGTAGCTATCACA-3′), and the negative control plasmid shRNA-NC (5′-TTCTCCGAACGTGTCACGT-3′) were obtained from GenePharma (Shanghai, China).

The DsRed-Mito was obtained from Clontech, and the mitophagy reporter plasmid pCLBW cox8-EGFP-mCherry (COX8-EGFP-mCherry) was obtained as a gift from Dr. David Chan (Addgene, #78520, Cambridge, MA, USA). Primary neurons were transfected using Lipofectamine 2000 (Invitrogen, Carlsbad, CA, USA) following the manufacturer’s instructions.

### 4.7. Western Blotting Analysis

Cell lysates were prepared, and Western blotting was performed as previously described [69]. Briefly, equal amounts of protein were separated using SDS-PAGE, transferred onto nitrocellulose membranes (Millipore, Billerica, MA, USA), and then blocked. The membranes were incubated with different primary antibodies, followed by the incubation with the HRP-conjugated secondary antibody. Protein bands were visualized using a FluorChem M Imaging System (ProteinSimple, San Jose, CA, USA).

### 4.8. Transmission Electron Microscopy (TEM)

TEM (Transmission Electron Microscopy) was performed following the previously described method [67,68]. Treated neurons were fixed in ice-cold 5% glutaraldehyde in 0.1 M sodium cacodylate buffer (pH 7.4) at 4 °C. After thoroughly rinsing with sodium cacodylate buffer, the cell pellets were further fixed on ice with 1% OsO4 in 0.1 M sodium cacodylate buffer. Subsequently, dehydration was carried out using a series of ethanol and acetone. The cell pellets were then embedded in resin and polymerized at 60 °C. Ultrathin sections were mounted onto copper grids and stained with 4% uranyl acetate and lead citrate. Imaging was performed using a transmission electron microscope (Hitachi, Tokyo, Japan).

### 4.9. Cell Viability Assay

Cell viability was assessed using a Cell Counting Kit-8 (FC101, TransGen Biotech, Beijing, China). Following the treatment, cells were incubated in a medium containing 10% CCK solution for 2 h. The absorbance at 450 nm was measured using a microplate reader, with a background control used as a blank. The cell survival ratio was calculated as the percentage of the untreated control.

### 4.10. Terminal Deoxynucleotidyl Transferase dUTP Nick End Labeling (TUNEL) Assay

TUNEL analysis was conducted to assess cellular apoptosis using an in situ cell death detection kit, TMR red (12156792910, Roche, Basel, Switzerland), following the manufacturer’s instructions. Primary cortical neurons were cultured on coverslips in a poly-D-lysine-coated 12-well plate at a density of 5 × 10^5^ cells/well. Cells were counterstained with propidium iodide (PI) for nuclei visualization. The slides were examined using an upright fluorescence microscope (Nikon, Tokyo, Japan).

### 4.11. Statistical Analysis

The data were presented as means ± standard deviation (SD) from three independent experiments. Parametric data were performed by one-way analysis of variance (ANOVA) with Turkey post hoc multiple comparisons using SPSS software (Version 21.0; SPSS Inc., Chicago, IL, USA). A *p*-value of <0.05 was considered statistically significant.

## Data Availability

The data for this study are available from the corresponding author upon reasonable request.

## Abbreviations

Abbreviations	Full Name
PrP^106–126^	PrP fragment 106–126
CNS	Central Nervous System
PINK1	PTEN-induced kinase 1
PHB2	Prohibitin 2
TOMM20	Translocase of the Outer Mitochondrial Membrane 20
LC3B	Light Chain 3B
DMEM	Dulbecco’s Modified Eagle’s Medium
PBS	Phosphate Buffer Saline
FBS	Fetal Bovine Serum
HBSS	Hank’s Balanced Salt Solution
SDS-PAGE	Sodium Dodecyl Sulfate-Polyacrylamide Gel Electrophoresis
DAPI	4,6-diamidina-2-phenylin
shRNA	Short hairpin RNA
TEM	Transmission Electron Microscopy
CCK	Cell Counting Kit
TUNEL	Terminal deoxynucleotidyl transferase dUTP nick end labeling
PI	Propidium Iodide
ANOVA	Analysis of Variance
SD	Standard Deviation
SPSS	Statistical Product and Service Solutions
